# Neuronal deletion of phosphatase and tensin homolog in mice results in spatial dysregulation of adult hippocampal neurogenesis

**DOI:** 10.3389/fnmol.2023.1308066

**Published:** 2023-12-07

**Authors:** Sarah E. Latchney, Brayan R. Ruiz Lopez, Paige D. Womble, Katherine J. Blandin, Joaquin N. Lugo

**Affiliations:** ^1^Department of Biology, St. Mary’s College of Maryland, St. Mary’s City, MD, United States; ^2^Department of Psychology and Neuroscience, Baylor University, Waco, TX, United States

**Keywords:** dentate gyrus, doublecortin, epilepsy, hippocampus, ki67, neurogenesis, proliferation and apoptosis, *Pten*

## Abstract

Adult neurogenesis is a persistent phenomenon in mammals that occurs in select brain structures in both healthy and diseased brains. The tumor suppressor gene, phosphatase and tensin homolog deleted on chromosome 10 (*Pten*) has previously been found to restrict the proliferation of neural stem/progenitor cells (NSPCs) *in vivo*. In this study, we aimed to provide a comprehensive picture of how conditional deletion of *Pten* may regulate the genesis of adult NSPCs in the dentate gyrus of the hippocampus and the subventricular zone bordering the lateral ventricles. Using conventional markers and stereology, we quantified multiple stages of neurogenesis, including proliferating cells, immature neurons (neuroblasts), and apoptotic cells in several regions of the dentate gyrus, including the subgranular zone (SGZ), outer granule cell layer (oGCL), molecular layer, and hilus at 4 and 10 weeks of age. Our data demonstrate that conditional deletion of *Pten* in mice produces successive increases in dentate gyrus proliferating cells and immature neuroblasts, which confirms the known negative roles *Pten* has on cell proliferation and maturation. Specifically, we observe a significant increase in Ki67+ proliferating cells in the neurogenic SGZ at 4 weeks of age, but not 10 weeks of age. We also observe a delayed increase in neuroblasts at 10 weeks of age. However, our study expands on previous work by providing temporal, subregional, and neurogenesis-stage resolution. Specifically, we found that *Pten* deletion initially increases cell proliferation in the neurogenic SGZ, but this increase spreads to non-neurogenic dentate gyrus areas, including the hilus, oGCL, and molecular layer, as mice age. We also observed region-specific increases in apoptotic cells in the dentate gyrus hilar region that paralleled the regional increases in Ki67+ cells. Our work is accordant with the literature showing that *Pten* serves as a negative regulator of dentate gyrus neurogenesis but adds temporal and spatial components to the existing knowledge.

## 1 Introduction

Aberrant neurogenesis within the adult hippocampal dentate gyrus is a characteristic feature in the development of temporal lobe epilepsy (Cho et al., 2015; Jessberger and Parent, 2015; Danzer, 2019; Lybrand et al., 2021). In healthy brains, inhibitory microcircuits within the dentate gyrus function as a gate, limiting excitatory activity throughout the hippocampus. In epileptic brains, however, it is believed that the abnormal production and integration of adult-born hippocampal neurons compromises the gating function of the dentate gyrus, leading to the spread of epileptic seizures (Bui et al., 2015; Jessberger and Parent, 2015; Danzer, 2019; Sparks et al., 2020; Lybrand et al., 2021). Cellular changes related to seizure-induced neurogenesis include the proliferation of neural stem/progenitor cells (NSPCs) (Shapiro et al., 2011; Cho et al., 2015), neuronal hypertrophy (Houser, 1990; Lurton et al., 1998; Backman et al., 2001; Kwon et al., 2001, 2003; Ogawa et al., 2007), sprouting of granule cell mossy fiber axons into the dentate gyrus molecular layer (Murphy et al., 2011; Du et al., 2017), the emergence of ectopic granule cells within the hilar region of the dentate gyrus (Pun et al., 2012; Lybrand et al., 2021), and the growth of atypical basal dendrites by granule cells (Shapiro et al., 2008; Murphy et al., 2012). These cellular abnormalities are thought to perturb dentate gyrus circuitry and heighten the excitability of the hippocampus, leading to spontaneous seizures (Morgan and Soltesz, 2008; Zhang et al., 2012; Jessberger and Parent, 2015; Danzer, 2019; Zhou et al., 2019).

At the molecular level, hyperactivation of the mammalian target of rapamycin (mTOR) signaling pathway has been observed in several models of epilepsy (Zeng et al., 2009; Huang et al., 2010; Pun et al., 2012; Zhang and Wong, 2012) and inhibition of mTOR reduces seizure activity (Zeng et al., 2009; Huang et al., 2010). Controlling the activity of this signaling pathway is the tumor suppressor phosphatase and tensin homolog on chromosome 10 (PTEN). PTEN negatively regulates the mTOR pathway and functions to block mTOR activity by deregulating molecules associated with this pathway, including phosphatidylinositol (3,4,5)-trisphosphate (PIP3) and phosphatidylinositol 4,5-bisphosphate (PIP2; Song et al., 2012). The actions of PTEN are critical for various processes associated with neurogenesis, including cell fate decisions, cell growth, axon formation, dendritic growth, and synaptogenesis (Bruel-Jungerman et al., 2009; Yu and Cui, 2016; LiCausi and Hartman, 2018) and loss of this molecule can lead to seizures (Backman et al., 2001; Kwon et al., 2001, 2003; Ogawa et al., 2007; Ljungberg et al., 2009; Santos et al., 2017).

Genetic mouse models using conditional deletion of *Pten* in NSPCs have shed light on the role of *Pten* in regulating the proliferation and self-renewing capacity of these cells (Sun et al., 1999; Groszer et al., 2001, 2006). In the embryonic brain, *Pten* deletion in NSPCs leads to an enlarged brain mass, primarily attributed to increased proliferation of NSPCs, reduced cell death, and enlarged cell size (Sun et al., 1999; Groszer et al., 2001, 2006). These early studies attribute the increased proliferation to decreased dependence on growth factors, enhanced G1 cell cycle entry, and abbreviated cell cycle duration (Sun et al., 1999; Groszer et al., 2001, 2006). In the adult brain, *Pten* deletion in NSPCs also results in increased proliferation of adult-born granule cells in the subventricular zone (SVZ) and accelerated their differentiation into mature neurons, resulting in an enlarged olfactory bulb and augmented olfactory function (Gregorian et al., 2009). Similarly, *Pten* deletion leads to increased proliferation and differentiation of NSPCs residing in the subgranular zone (SGZ) of the adult hippocampal dentate gyrus (Amiri et al., 2012), thereby increasing susceptibility to temporal lobe seizures (Ogawa et al., 2007; Amiri et al., 2012; Pun et al., 2012; Santos et al., 2017).

While it is evident that loss of *Pten* and subsequent activation of mTOR is sufficient to increase the generation and integration of adult-born granule cells in the hippocampus, there remain unanswered questions. First, the neurogenesis data observed by Amiri et al. (2012) are reported as an estimate of cell density (e.g., number of cells per section) that was obtained from two-dimensional images rather than an approximation of the total number of cells. Estimates of numerical density are biased because the probability that a cell appears in a section is related to its size, shape, and orientation. Moreover, when the number of cells is reported as per unit volume of tissue, there is no information about the volume of the structure (West and Gundersen, 1990). This is especially critical given the structural malformations in *Pten*^–/–^ mice (Backman et al., 2001; Kwon et al., 2001, 2003). Second, because afferent and efferent connections and hippocampal function vary along its septotemporal axis (Wu and Hen, 2014; Wu et al., 2015; Bonaguidi et al., 2016; Wiget et al., 2017; Ayhan et al., 2021), obtaining an estimate of absolute cell number is necessary to understand how neurogenesis may be affected along the hippocampal septotemporal axis. Third, as explained above, seizures not only stimulate hippocampal neurogenesis but some of the newly generated neurons are generated outside of the neurogenic SGZ, particularly in the hilus [reviewed in Kasahara et al. (2023)]. While one study demonstrated that deletion of *Pten* in 14-day-old mice leads to an increase in ectopic granule cells (Pun et al., 2012), there has yet to be a study that comprehensively quantifies the number of adult-generated neurons in various subfields of the adult dentate gyrus, beyond the hilus. Lastly, there is a scarcity of studies that examine sex differences in *Pten*-deficient mice, as studies to date have either only focused on male mice or do not report detailed sex analysis of their data.

To fill these knowledge gaps, we performed a detailed analysis of adult dentate gyrus neurogenesis in 4 and 10 week-old male and female wild-type and *Pten*^–/–^ mice. Because dentate gyrus neurogenesis is a multi-step process that occurs in phases – including the proliferation of neuronal stem cells and progenitors in the SGZ, their differentiation into neuroblasts, and subsequent survival into glutamatergic granule cells (Aimone et al., 2014; Bond et al., 2015; Kempermann et al., 2015) – we quantified multiple neurogenic markers to evaluate the proliferation, differentiation, and apoptosis of adult-born granule cells. Using unbiased stereological quantification, we analyzed measures of proliferation (Ki67+ cells), neuroblasts (DCX+ cells), and apoptosis [cleaved caspase-3 + (CC3+ ) cells] in the neurogenic SGZ layer as well as ectopic locations, including the hilus, outer granule cell layer, and the molecular layer. Our comprehensive analysis reveals that the increase in neurogenesis in adult *Pten*^–/–^ mice initially begins in the SGZ neurogenic zone at 4 weeks of age but spreads to ectopic locations at 10 weeks of age. We also explore differences along the septotemporal axis of the hippocampus and report sex-dependent differences in our data.

## 2 Materials and methods

### 2.1 Mice

**FIGURE 1:**
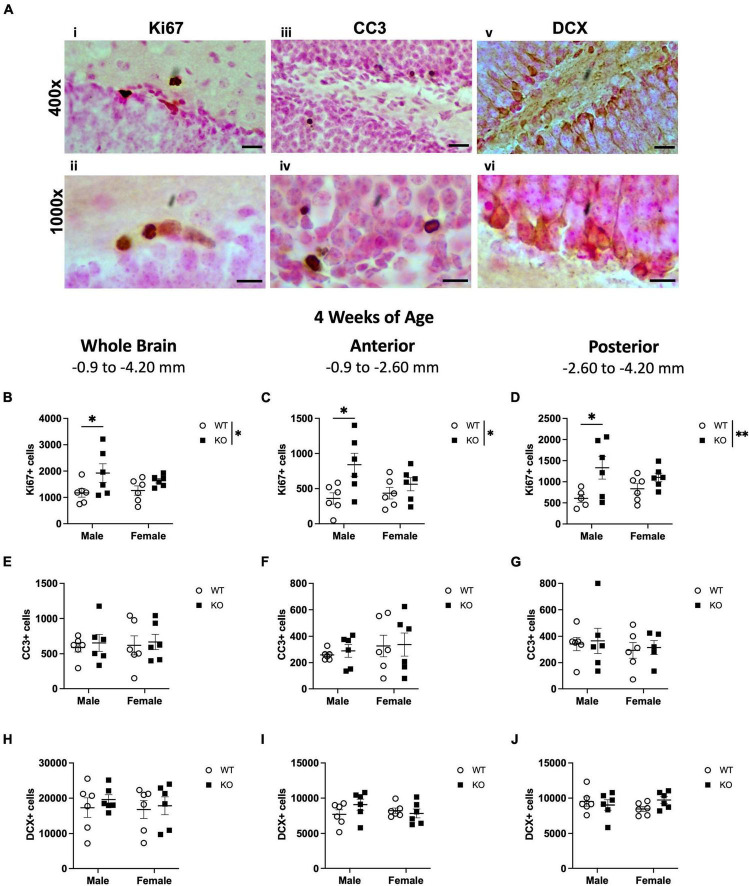
Male *Pten*^–/–^ mice have more Ki67-immunoreactive (Ki67+) proliferating cells in the subgranular zone (SGZ) at 4 weeks of age. Representative photomicrographs of Ki67 **(Ai,ii)**, CC3 **(Aiii,iv)**, and DCX **(Av,vi)**-stained tissue. Top images = 400x magnification. Scale bar = 20 μm. Bottom images = 1,000x magnification. Scale bar = 10 μm. Stereological quantification of Ki67+ **(B–D)**, CC3+ **(E–G)**, and DCX+ **(H–J)** cells in the SGZ (Ki67 and CC3) and SGZ/GCL (DCX). Immunopositive cells were quantified considering immunopositive cells in the SGZ across the entire septotemporal axis, and also divided into anterior dentate gyrus and posterior dentate gyrus, operationally defined as Bregma levels –0.90 to –2.60 and –2.60 to –4.20, respectively. 2-way ANOVA, *p < 0.05 and **p < 0.01. *n* = 5–6 per group.

Mice used in this study were male and female adult neuron subset-specific *Pten* (NS-*Pten*) conditional mice, previously described as GFAP-Cre; Pten^loxP/loxP^ mice (RRID: MGI:3714016; Hodges et al., 2018, 2021). This mouse model has Cre activity mainly in hippocampal granule neurons of the dentate gyrus (Backman et al., 2001; Kwon et al., 2001). Previous studies have repeatedly confirmed that the Cre-mediated expression of β-galactosidase is specific to NeuN-positive granule neurons in the cerebellum and the dentate gyrus (see Figure 1 in both Backman et al., 2001; Kwon et al., 2001). In the subventricular zone, all Cre-expressing cells also express GFAP and Nestin, characteristics of neural stem/progenitor cells (Gregorian et al., 2009). In contrast to neurons, Cre activity was rarely detected in S100β and GFAP-positive glial cells [see Figure 1B in Kwon et al. (2001)]. We have been breeding *NS-Pten*^*loxP*/+^ mice on an FVB-based mixed background strain for more than 10 generations and used heterozygote parents to produce *NS-Pten^+/+^* wildtype (WT), *NS-Pten*^*loxP*/+^ heterozygous (HT), and *Pten*^loxP/loxP^ knockout (KO). Because *Pten* heterozygous mice do not display deficits in learning and memory or in any social, repetitive, or locomotor behaviors (Smith et al., 2016), only homozygous WT and KO mice were used in this study. All mice were bred and housed as cage mates at Baylor University under standard laboratory conditions with an ambient temperature of 22°C and a 12°h light/dark diurnal cycle. Mice were provided with water and food *ad libitum*. All procedures were conducted in compliance with the Baylor University Institutional Animal Care and Use Committee and the *National Institutes of Health Guidelines for the Care and Use of Laboratory Animals* (Hodges et al., 2018, 2021).

### 2.2 Tissue collection and immunohistochemistry (IHC)

Mice were anesthetized using isoflurane prior to intracardial perfusion with 4°C 0.1 M phosphate-buffered saline (PBS) containing 2 IU/mL heparin for exsanguination followed by 4% paraformaldehyde for fixation. Brains were extracted and immersed in 4% paraformaldehyde in 0.1 M PBS at 4°C for 24 h followed by cryoprotection in 0.1 M PBS with 30% sucrose and 0.01% sodium azide. The brain of each mouse was sectioned in the coronal plane extending from anterior to the lateral ventricles to the cerebellum (1.70 to −4.20 mm from Bregma) was sectioned at 30 μm using a cryostat (Thermo Scientific HM 525NX Cryostat) in a 1:8 series to permit stereological quantification. Brain sections were stored in cryoprotectant at −20°C until IHC.

Slide-mounted IHC for immunopositive cells in the dentate gyrus [Ki67+ , doublecortin (DCX)+ , and cleaved caspase-3 (CC3)+ cells] and the subventricular zone (Ki67+ and CC3+ cells) was performed as previously described (Latchney et al., 2014, 2015). For each IHC procedure, one entire series containing the hippocampus (−0.9 to −4.20 mm from Bregma; every 8th section) or lateral ventricles (1.70 to −0.90 mm from Bregma; every 8th section) was slide-mounted onto Superfrost-Plus charged slides (ThermoFisher Scientific, 12-550-16, Pittsburgh, PA, United States) and allowed to dry for 2 h. We performed antigen retrieval on slide-mounted sections (0.01 M citric acid pH 6.0, 90−95°C, 15 min) followed by washing in room temperature 1x PBS. For CC3 IHC, two additional antigen retrieval steps were performed: permeabilization (0.1% Trypsin in 0.1 M Tris and 0.1% CaCl_2_, 10 min) and denaturation (2N HCl in 1x PBS, 30 min). Endogenous peroxidase activity was inhibited via incubation with 0.3% hydrogen peroxide (H_2_O_2_) for 30 min. Non-specific binding was blocked with 3% serum (goat) and 0.3% Triton-X 100 in 1x PBS for 60 min. The sections were then incubated with the appropriate primary antibody in 3% serum and 0.3% Tween-20 overnight. The following primary antibodies were used: rabbit-anti-Ki67 (1:500; Thermo Fisher Scientific, MA514520, Freemont, CA, United States), guinea pig-anti-DCX (1:500; Millipore, AB2253, Billerica, MA, United States), and rabbit-anti-cleaved caspase-3 (1:500; Cell Signaling, 9661S). Primary antibody incubation was followed by 1x PBS rinses and incubation with biotinylated secondary antibodies (goat-anti-rabbit-IgG, 111-065-003; goat-a-guinea pig-IgG, 106-065-003; all 1:200 and from Jackson ImmunoResearch, West Grove, PA, United States) for 2 h. After additional 1x PBS rinses, slides were incubated with an avidin-biotin complex for 90 min (Elite ABC-HRP Kit, PK-6100, Vector Laboratories, Burlingame, CA, United States). After another set of rinses in 1x PBS, immunoreactive cells were visualized via incubation with 3,3′-diaminobenzidine metal-concentrate (Thermo Fisher Scientific, PI34065, Pittsburgh, PA, United States) for ∼10 min. Slides were counterstained with Nuclear Fast Red (Vector Laboratories, H-3403-500). We then performed a series of increasing ethanol concentrations to dehydrate the sections and coverslipped using DPX mountant (Thermo Fisher Scientific, 50-980-370, Pittsburgh, PA, United States).

### 2.3 Cavalieri volume estimation

Estimation of granule cell layer (GCL) volume was quantified using the Cavalieri Probe within the StereoInvestigator software (Gundersen et al., 1988; Latchney et al., 2014, 2015; Basler et al., 2017). Every 8th section was analyzed to assess the volume of the GCL. All measurements were acquired using the Stereo Investigator software (MBF Bioscience, Williston, VT, United States) at 100x magnification (10x objective, NA 0.30) on a Zeiss AxioImager M2 microscope. Area sizes were determined with the area measurement tool in which a grid of test points was overlaid over a live image of each section that contained the dentate gyrus. The area of the GCL was estimated from the total number of points that fell within the GCL. The area of the GCL was calculated at each distance from Bregma. To obtain the volume, the sum of the area measured was multiplied by the section sampling fraction (8) and the section thickness (30 μm) (Gundersen and Jensen, 1987; Gundersen et al., 1988; Latchney et al., 2014, 2015). The Gunderson coefficient of variance for each mouse was always <10% (Latchney et al., 2014, 2015). Data are reported as the total estimated volume of the GCL per brain (in cubic millimeters).

### 2.4 Cell type quantification

Unbiased stereology was used to enumerate Ki67, CC3, and DCX immunopositive cells. Due to their rarity (West and Gundersen, 1990; Latchney et al., 2014, 2015), Ki67+ and CC3+ cells were quantified in every 8th section throughout the subventricular zone lining the lateral ventricles (Bregma levels 1.7 to −1.9) and the entire hippocampus (Bregma levels −0.90 to −4.20). Cells were visualized with a Nikon Eclipse 80i microscope at 400x magnification (40X objective; NA 0.75) with continuous adjustment through the depth of the section. Characteristics that were taken into consideration when determining Ki67+ or CC3+ cells were size, color, shape, transparency, location, and focal plane (Latchney et al., 2014, 2015; Clark et al., 2020). As described in Clark et al. (2020), Ki67+ and CC3+ cells were enumerated in four areas of the dentate gyrus: the SGZ (30 μm into the hilus and the inner half of the GCL), frequently considered to be the “neurogenic niche” of the dentate gyrus (Riquelme et al., 2008; Zhao et al., 2008; Obernier and Alvarez-Buylla, 2019); the outer GCL (oGCL), to which a small number of adult-generated cells migrate (Kempermann et al., 2003); the hilus, through which dentate gyrus granule cells extend their processes toward CA3; and the molecular layer, where the dendrites of dentate gyrus granule cells are located.

Total cell counts were multiplied by a section sampling fraction (ssf) of 8 to attain an estimate of the total cell number. Because initial cell counts (before multiplication) for immunopositive cells were low according to dissector/fractionator standards, the area sampling fraction (asf) and height sampling fraction (hsf) were both set to 1, as is commonly used for counting rare cell populations (West and Gundersen, 1990; Latchney et al., 2014, 2015). All cell quantification was performed blindly.

DCX+ cells were quantified via unbiased stereology in the SGZ and the entire GCL. DCX+ cells in the hilus and molecular layer appeared faint and ill-defined and were not enumerated. Because DCX+ cells in the SGZ and GCL are expressed in high numbers compared to the relatively sparse numbers of Ki67+ and CC3+ cells, DCX+ cells in the SGZ and GCL were quantified using the optical fractionator procedure in Stereo Investigator (MicroBrightField, MBF) (Latchney et al., 2014, 2015; Clark et al., 2020). Every 8th coronal section throughout the hippocampus was analyzed on a Zeiss AxioImager M2 microscope. The SGZ and GCL were outlined for each section at 100X magnification (10X objective, NA 0.30). An unbiased counting frame overlaid on the region of interest was used to enumerate immunopositive cells. Quantification was performed at 400X (40X objective, NA 0.75) by focusing throughout the depth of the section. At least 200 cells per mouse were counted and the average number of counting fields per mouse was close to 300 in an average of 10 sections per mouse (Latchney et al., 2014, 2015). DCX+ cells were counted if they satisfied three criteria: the entire border of the cell body was intact, there was a dendritic process emerging from the cell body, and the cell body was darker than the surrounding background (Latchney et al., 2014, 2015; Clark et al., 2020). To decrease the effect of shrinkage on the tissue, the average measured mounting thickness after processing was maintained at ∼20 μm, and we used an optical dissector height of 12 mm. The area-sampling fraction (asf) was 1/8 and every 8th section (section sampling fraction; ssf) was used to obtain a total estimate of the number of DCX+ cells within the SGZ and GCL. The Gunderson coefficient of variance for each mouse quantified was always <10% (Latchney et al., 2014, 2015). Data are reported as the total number of DCX+ cells per brain. All quantification was performed blindly.

### 2.5 Quantification of Ki67+, CC3+, and DCX+cells along the septotemporal axis

For anterior and posterior analysis of Ki67+, CC3+, and DCX+ immunopositive cells, results were split into anterior and posterior regions, demarcated by Bregma level −2.60 mm (Tanti et al., 2012; Tanti and Belzung, 2013; Zhou et al., 2016; Clark et al., 2020). Bregma level −2.60 mm is when hippocampal CA3 reaches halfway through the dorsal-ventral extent of the brain, and the corpus callosum no longer joins the two hemispheres (Paxinos and Franklin, 2019). Counts for each section anterior to −2.60 mm were summated and multiplied by 8 to obtain an anterior value for each mouse. The same procedure was used for the posterior analysis, using all sections posterior to Bregma level −2.60 mm.

### 2.6 Data presentation and analysis and image presentation

**FIGURE 2:**
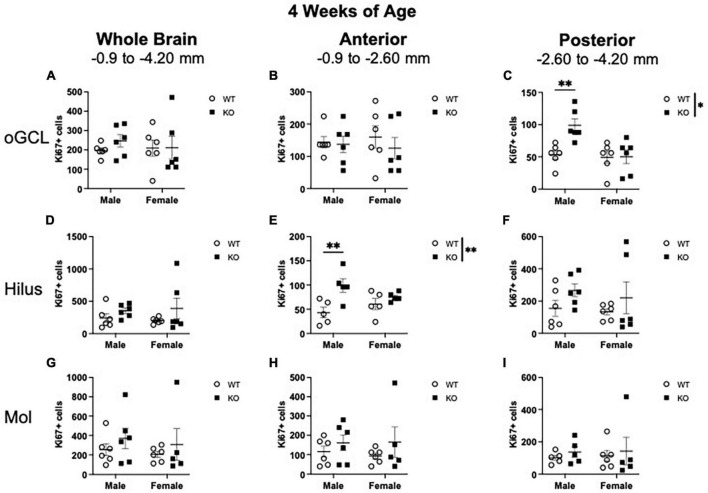
Male *Pten*^–/–^ mice exhibit select increases in Ki67+ proliferating cells in the posterior dentate gyrus outer granule cell layer (oGCL) and anterior hilus at 4 weeks of age. Stereological quantification of Ki67+ (*n* = 5–6 per group) cells in the oGCL **(A–C)**, hilus **(D–F)**, and molecular layer (Mol; **G**–**I**). Immunopositive cells were quantified across the entire septotemporal axis **(A,D,G)**, and also broken up into anterior **(B,E,H)**, and posterior **(C,F,I)** bins, operationally defined as Bregma levels –0.90 to –2.60 and –2.60 to –4.20, respectively. 2-way ANOVA, *p < 0.05 and **p < 0.01.

**FIGURE 3:**
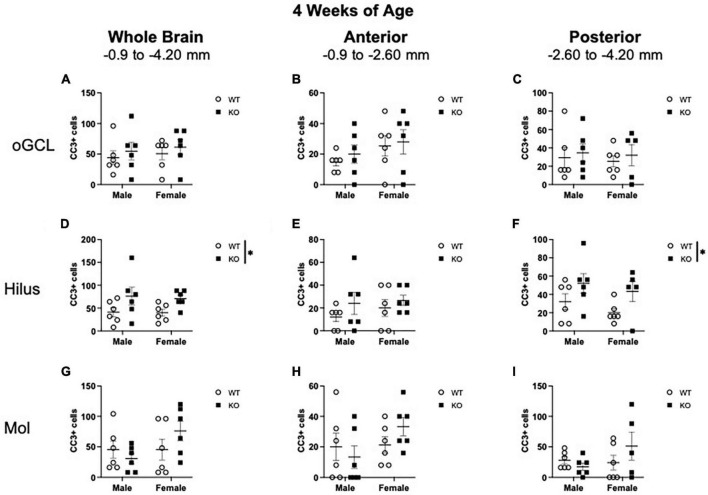
*Pten*^–/–^ mice exhibit select increases in cleaved caspase-3-immunoreactive (CC3+) cells in the dentate gyrus hilar region at 4 weeks of age. Stereological quantification of CC3+ (*n* = 5–6 per group) cells in the oGCL **(A–C)**, hilus **(D–F)**, and molecular layer (Mol; **G–I**). Immunopositive cells were quantified across the entire septotemporal axis **(A,D,G)**, and also broken up into anterior **(B,E,H)**, and posterior **(C,F,I)** bins, operationally defined as Bregma levels −0.90 to −2.60 and −2.60 to −4.20, respectively. 2-way ANOVA, *p < 0.05.

**FIGURE 4:**
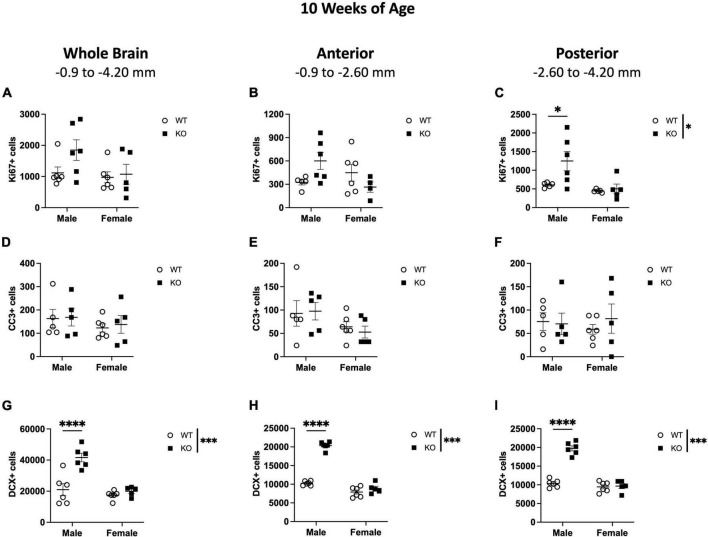
Male *Pten*^–/–^ mice have more DCX-immunoreactive (DCX+) cells in the SGZ/GCL at 10 weeks of age. Stereological quantification of Ki67+ **(A–C)**, CC3+ **(D–F)**, and DCX+ **(G–I)** cells in the SGZ (Ki67 and CC3) and SGZ/GCL (DCX). Immunopositive cells were quantified considering immunopositive cells in the SGZ across the entire septotemporal axis, and also divided into anterior dentate gyrus and posterior dentate gyrus, operationally defined as Bregma levels –0.90 to –2.60; and –2.60 to –4.20, respectively. 2-way ANOVA, *p < 0.05, ***p < 0.001, and ****p < 0.0001. *n* = 5–6 per group.

**FIGURE 5:**
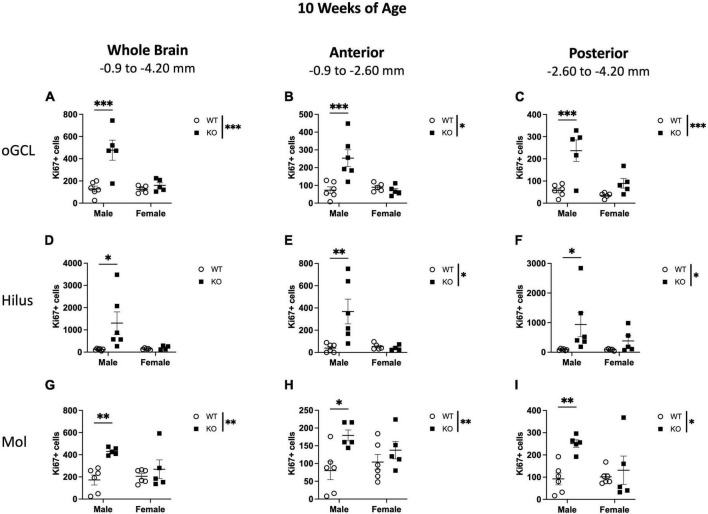
Male *Pten*^–/–^ mice have more Ki67+ proliferating cells in the dentate gyrus outer granule cell layer (oGCL), hilus, and molecular layer (Mol) at 10 weeks of age. Stereological quantification of Ki67+ (*n* = 5–6 per group) cells in the oGCL **(A–C)**, hilus **(D–F)**, and Mol layer **(G–I)**. Immunopositive cells were quantified across the entire septotemporal axis **(A,D,G)**, and also broken up into anterior **(B,E,H)**, and posterior **(C,F,I)** bins, operationally defined as Bregma levels –0.90 to –2.60 and –2.60 to –4.20, respectively. 2-way ANOVA, *p < 0.05, **p < 0.01, and ***p < 0.001.

**FIGURE 6:**
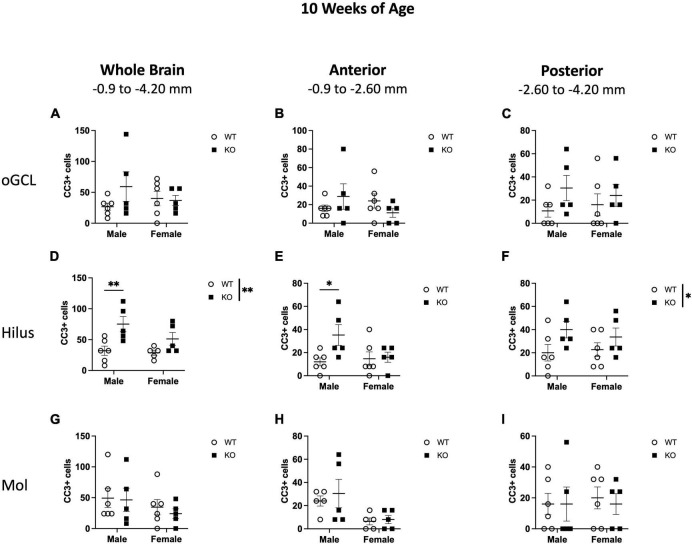
*Pten*^–/–^ mice exhibit select increases in cleaved caspase-3-immunoreactive (CC3+) cells in the dentate gyrus hilar region at 10 weeks of age. Stereological quantification of CC3+ (*n* = 5–6 per group) cells in the oGCL **(A–C)**, hilus **(D–F)**, and molecular layer (Mol; **G–I**). Immunopositive cells were quantified across the entire septotemporal axis **(A,D,G)**, and also broken up into anterior **(B,E,H)**, and posterior **(C,F,I)** bins, operationally defined as Bregma levels –0.90 to –2.60 and –2.60 to –4.20, respectively. 2-way ANOVA, *p < 0.05 and **p < 0.01.

**FIGURE 7:**
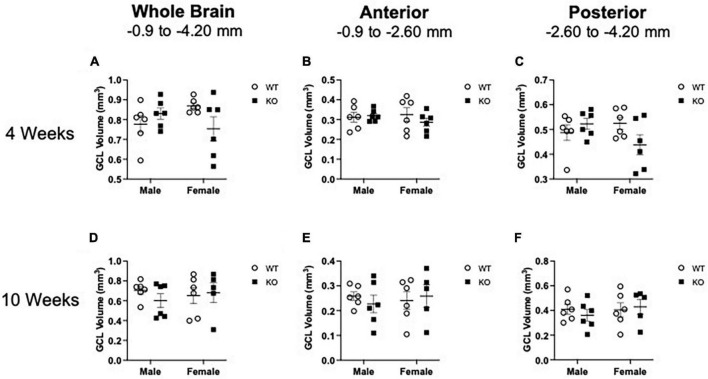
Mean granule cell layer (GCL) volume in WT and *Pten*^–/–^ mice at 4 and 10 weeks of age (shown in mm^3^). Stereological quantification of GCL volume at 4 weeks **(A–C)** and 10 weeks **(D–F)** of age. GCL volume was quantified across the entire septotemporal axis and also divided into anterior dentate gyrus and posterior dentate gyrus, operationally defined as Bregma levels –0.90 to –2.60 and –2.60 to –4.20, respectively. 2-way ANOVA. *n* = 5–6 per group.

**FIGURE 8:**
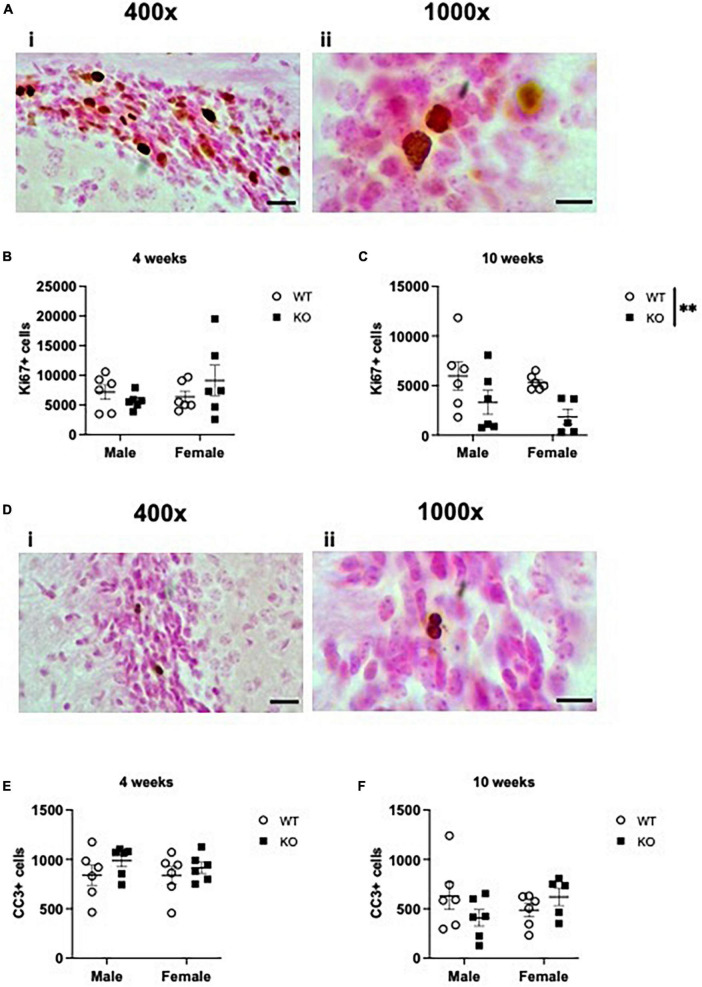
*Pten*^–/–^ mice have fewer Ki67-immunoreactive (Ki67+) proliferating cells in the subventricular zone (SVZ) lining the lateral ventricles at 10 weeks of age. Representative photomicrographs of Ki67-stained tissue [**(Ai)**: 400x magnification. Scale bar = 20 μm; **(Aii)**: 1,000x magnification. Scale bar = 10 μm]. Stereological quantification of Ki67+ cells in the SVZ at 4 weeks **(B)** and 10 weeks **(C)** of age. Representative photomicrographs of CC3-stained tissue [**(Di)**: 400x magnification. Scale bar = 20 μm; **(Dii)**: 1,000x magnification. Scale bar = 10 μm]. Stereological quantification of CC3+ cells in the SVZ at 4 weeks **(E)** and 10 weeks **(F)** of age. All immunopositive cells were quantified across the entire septotemporal axis (Bregma levels 1.8 to –0.9). 2-way ANOVA, **p < 0.01. *n* = 5–6 per group.

Experimenters were blinded and code was broken after completion of data analyses. Because sex differences were not a primary research question, but the inclusion of sex as a biological variable has become standard in research designs, analyses, and reporting (Clayton and Collins, 2014), we originally designed the study to have 10−12 mice per genotype with equal numbers of male and female mice. However, preliminary analysis indicated that there could be a sex-specific trend in our findings. For that reason, male and female mice were separated so that Figures 1–8 display four comparison groups with 5−6 mice per group: Male WT, Male KO, Female WT, and Female KO. Data are displayed as individual data points with mean and standard error of the mean. All data were assessed for normality with the Shapiro-Wilk test. The Grubbs Outlier test (GraphPad Prism) was used to check for and exclude statistical outliers. Statistical analyses were performed with 2-way ANOVA and graphs were generated in GraphPad Prism (version 9.0). Sidak’s *post-hoc* comparisons were used to analyze significant 2-way ANOVAs. *P* ≤ 0.05 denoted statistical significance. Complete details of ANOVA analyses for each figure panel are provided in Supplementary Tables 1–4. In the same Supplementary Tables 1–4, we also provide complete statistical details for the combined-sex analyses. For combined-sex analyses, normal data were analyzed with unpaired two-tailed *t*-test, and non-normal data were analyzed with the Mann-Whitney test. Images were taken with a Zeiss Axiocam 305 camera using Zeiss Zen Blue software and imported into ImageJ for labeling. Representative images taken at 400x and 1000x magnification are provided in Figures 1–8.

## 3 Results

While previous mouse models of *Pten* deletion had demonstrated a role of *Pten* in NSPC proliferation and differentiation in the hippocampus (Kwon et al., 2001, Kwon et al., 2006; Amiri et al., 2012), cortex (Groszer et al., 2001, 2006), and the subventricular zone (SVZ; Gregorian et al., 2009), we expand on these studies by using a more comprehensive method of quantifying adult neurogenesis. Here, we used stereology to quantify various indices of neurogenesis across the entire extent of the dentate gyrus and the SVZ. In addition to the well-established neurogenic locations (SGZ and SVZ), we also quantified proliferating cells in non-neurogenic regions of the dentate gyrus. Given that *Pten* negatively regulates neurogenesis (Groszer et al., 2001, 2006; Gregorian et al., 2009; Amiri et al., 2012) and *Pten*^–/–^ mice develop more seizures compared to wild-type mice (Ogawa et al., 2007; Amiri et al., 2012; Santos et al., 2017), we hypothesized that deletion of *Pten* would result in increased levels of proliferation and differentiation of adult-born cells. To assess cells in stages of dentate gyrus neurogenesis, we enumerated the number of proliferating precursors (Ki67+ cells), neuroblasts (DCX+ cells), and apoptotic cells (CC3+ cells) at 4 and 10 weeks of age. Ki67+ and CC3+ cells were also quantified in the SVZ lining the lateral ventricles. Below, we discuss our results and provide full details of statistical analyses in Supplementary Tables 1–4.

### 3.1 Neurogenesis indices at 4 weeks of age in the dentate gyrus neurogenic regions

Ki67 is an endogenous marker that is expressed in cells undergoing all phases of the cell cycle, including G1, S, G2, and M. Therefore, Ki67+ cells are considered to be “proliferating” (Mandyam et al., 2007). Ki67+ cells were evident in the SGZ of both WT and KO mice, appearing as dense nuclei smaller than 10 μm in diameter (Figures 1Ai, ii). In the SGZ at 4 weeks of age, stereological quantification revealed no interaction or effect of sex but did show an effect of genotype (F_1,20_ = 6.658; *p* = 0.018), indicating a 46% increase in Ki67+ nuclei in KO mice compared to WT mice (Figure 1B). Male, but not female, KO mice also demonstrated a ∼64% increase in Ki67+ nuclei compared to male WT (Sidak’s *post-hoc*; *p* = 0.048). Because synaptic connections and hippocampal function vary along the septotemporal axis (Wu and Hen, 2014; Wu et al., 2015; Bonaguidi et al., 2016; Wiget et al., 2017; Ayhan et al., 2021), Ki67+ cells were also enumerated in the anterior and posterior SGZ (Tanti and Belzung, 2013; Clark et al., 2020). Similar to the analysis on the entire SGZ (Figure 1B), there was a main effect of genotype in the anterior (F_1,20_ = 7.617; *p* = 0.012; Figure 1C) and posterior (F_1,19_ = 8.695; *p* = 0.008; Figure 1D) SGZ at 4 weeks of age but no interaction or effect of sex. KO mice exhibited a 76 and 69% increase in the anterior and posterior SGZ, respectively, compared to WT mice. Male, but not female, KO mice demonstrated a 133% increase in Ki67+ nuclei in the anterior SGZ (Sidak’s *post-hoc*; *p* = 0.012) and a 120% increase in the posterior SGZ (Sidak’s *post-hoc*; *p* = 0.016) compared to male WT.

The continuous production of adult-generated neurons is offset by ongoing cell death and is essential for regulating neurogenesis (Sierra et al., 2010; Beccari et al., 2017). It is possible that an increase in cell death could also accompany the increase in Ki67+ cells in the SGZ. To investigate this, we quantified cell death using cleaved caspase-3 (CC3) to label apoptotic cells. As expected, CC3+ cells were evident in the dentate gyrus SGZ of both WT and KO mice as punctate nuclei (Figures 1Aiii, iv). In the SGZ at 4 weeks of age, stereological quantification revealed no effect of genotype, sex, or interaction on the number of CC3+ cells (Figure 1E). There was also no interaction or effect of genotype or sex in CC3+ cell number in either the anterior (Figure 1F) or posterior (Figure 1G) SGZ.

The SGZ and GCL of WT and KO mice were examined for cells expressing DCX, a microtubule-associated protein expressed in neuroblasts (Francis et al., 1999; Nacher et al., 2001; Couillard-Despres et al., 2005; La Rosa et al., 2019). Neurogenic DCX+ cells were clearly distinguishable and densely populated the SGZ and GCL (Figures 1Av, vi). In the SGZ and GCL of WT and KO mice at 4 weeks of age, there was no interaction or effect of genotype or sex in the total DCX+ cell number (Figure 1H). When the SGZ and GCL in WT and KO mice were separated into anterior and posterior regions, there was also no interaction or effect of genotype or sex in DCX+ cell number in either the anterior (Figure 1I) or posterior (Figure 1J) SGZ and GCL. These data show that in the main neurogenic SGZ region at 4 weeks of age, male KO mice demonstrate an increase in Ki67+ proliferating cells but not in DCX+ neuroblasts. The increase in cell proliferation was not accompanied by an increase in apoptosis.

### 3.2 Neurogenesis indices at 4 weeks of age in the dentate gyrus non-neurogenic regions

The SGZ of the GCL is considered to be the main dentate gyrus neurogenic region. It is rare for other subregions of the dentate gyrus, including the oGCL, hilus, and molecular layer, to produce new neurons. However, these “non-neurogenic” subregions can contain proliferating cells (Garcia-Martinez et al., 2020). Therefore, the oGCL, hilus, and molecular layer from WT and KO mice were evaluated for Ki67+ immunoreactivity. For DCX+ analysis, DCX+ cells were analyzed in the SGZ and GCL (Clark et al., 2020).

In the oGCL, stereological quantification revealed no interaction or effect of genotype or sex for the total number of Ki67+ cells (Figure 2A). Parcellation of WT and KO Ki67+ oGCL cell counts into anterior and posterior dentate gyrus regions revealed a significant interaction (F_1,20_ = 5.942; *p* = 0.024) and effects of genotype (F_1,20_ = 6.299; *p* = 0.021) and sex (F_1,20_ = 8.242; *p* = 0.009) in the posterior (Figure 2C) but not anterior (Figure 2B) oGCL. In the posterior oGCL, male KO mice had ∼86% more Ki67+ cells compared to male WT mice, while female WT and KO mice exhibited similar numbers of Ki67+ (Sidak’s *post-hoc*; *p* = 0.005; Figure 2C). In the hilus, there was no interaction or individual effects of genotype or sex for the total number of Ki67+ cells (Figure 2D). When divided into anterior and posterior regions, the anterior hilus exhibited a significant main effect of genotype (F_1,16_ = 10.85; *p* = 0.005) but no interaction or effect of sex (Figure 2E). In the anterior hilus, male KO mice had 129% more Ki67+ cells compared to male WT mice, while female WT and KO mice exhibited similar numbers of Ki67+ (Sidak’s *post-hoc*; *p* = 0.004; Figure 2E). The posterior hilus did not exhibit an interaction or main effects of genotype or sex (Figure 2F). In the molecular layer, there was no interaction or main effects of genotype or sex for the number of Ki67+ cells spanning the entire hippocampus (Figure 2G) or when divided into anterior (Figure 2H) or posterior (Figure 2I) regions.

When CC3+ cells were quantified in the oGCL (Figures 3A–C), hilus (Figures 3D–F), and Mol layer (Figures 3G–I), only the hilus displayed a significant effect of genotype (F_1,20_ = 7.02; *p* = 0.015; Figure 3D). No main effect of sex or interaction was observed in this subregion. When divided into anterior and posterior regions, only the posterior hilus exhibited a significant main effect of genotype (F_1,19_ = 5.73; *p* = 0.027; Figure 3F). *Post-hoc* analyses did not reveal significant sex differences.

Taken together, these results show that at 4 weeks of age, the increased proliferation in the dentate gyrus of *Pten*-null KO mice is largely confined to the neurogenic SGZ (Figures 1B–D) and does not occur in other areas that are not typically thought to be neurogenic. While apoptosis seemed to be elevated in the hilus region of KO mice, cell death was not widespread throughout the hippocampus.

### 3.3 Neurogenesis indices at 10 weeks of age in the dentate gyrus neurogenic regions

The same markers examined at 4 weeks of age were also analyzed at 10 weeks of age. At 10 weeks of age, stereological quantification of total Ki67+ cells in the SGZ revealed no interaction or effect of genotype or sex (Figure 4A). When the SGZ in WT and KO mice were separated into anterior and posterior regions, there was an interaction (F_1,17_ = 6.019; *p* = 0.025) in the anterior SGZ but no effect of genotype or sex (Figure 4B). In the posterior SGZ, there was no interaction, but there was a main effect of genotype (F_1,17_ = 4.549; *p* = 0.048) and sex (F_1,17_ = 7.667; *p* = 0.013; Figure 4C), indicating a 105% increase in Ki67+ nuclei in male KO mice compared to male WT mice (Sidak’s *post-hoc*; *p* = 0.023; Figure 4C). No difference was observed between female WT and KO mice.

Stereological quantification of CC3+ cells in the SGZ at 10 weeks of age revealed no effect of genotype, sex, or interaction on the number of CC3+ cells (Figure 4D). When the SGZ in WT and KO mice were separated into anterior and posterior regions, there was also no interaction or effect of genotype or sex in CC3+ cell number in either the anterior (Figure 4E) or posterior (Figure 4F) SGZ.

In the SGZ and GCL of WT and KO mice at 10 weeks of age, there was a significant interaction (F_1,19_ = 12.0; *p* = 0.003) and effects of genotype (F_1,19_ = 19.50; *p* < 0.001) and sex (F_1,19_ = 23.29; *p* < 0.001) in the number of DCX+ cells across the entire dentate gyrus (Figure 4G). When the SGZ and GCL were separated into anterior and posterior regions, there was also significant interaction (Anterior: F_1,19_ = 93.96; *p* < 0.0001; Posterior: F_1,19_ = 60.34; *p* < 0.0001) and main effects of genotype (Anterior: F_1,19_ = 137.6; *p* < 0.0001; Posterior: F_1,19_ = 65.68; *p* < 0.0001) and sex (Anterior: F_1,19_ = 215.6; *p* < 0.0001; Posterior: F_1,19_ = 86.08; *p* < 0.0001) in DCX+ cells in both the anterior (Figure 4H) and posterior (Figure 4I) SGZ and GCL. Male KO mice demonstrated a 98% increase in total DCX+ cells in the SGZ and GCL (Sidak’s *post-hoc*; *p* < 0.0001), a ∼98% increase in DCX+ cells in the anterior SGZ and GCL (Sidak’s *post-hoc*; *p* < 0.0001), and a ∼90% increase in the posterior SGZ and GCL (Sidak’s *post-hoc*; *p* < 0.0001) compared to WT mice. These data show that at 10 weeks of age, male KO mice largely demonstrate an increase in DCX+ neuroblasts, but not in proliferating Ki67+ cells, in the SGZ neurogenic region.

### 3.4 Neurogenesis indices at 10 weeks of age in the dentate gyrus non-neurogenic regions

In the oGCL, stereological quantification revealed a significant interaction (F_1,17_ = 10.95; *p* = 0.004) and main effects of genotype (F_1,17_ = 16.41; *p* = 0.001) and sex (F_1,17_ = 11.40; *p* = 0.003) for the total number of Ki67+ cells (Figure 5A). KO mice demonstrated a 152% increase in the total number of Ki67+ compared to WT, with male KO mice exhibiting a ∼273% increase compared to WT males (Sidak’s *post-hoc*; *p* = 0.0001; Figure 5A). Female WT vs. KO mice did not demonstrate significant differences. Parcellation of WT and KO Ki67+ oGCL cell counts into anterior and posterior dentate gyrus regions also revealed a significant interaction (Anterior: F_1,18_ = 11.57; *p* = 0.003; Posterior: F_1,17_ = 5.680; *p* = 0.029) and main effects of genotype (Anterior: F_1,18_ = 7.537; *p* = 0.0133; Posterior: F_1,17_ = 19.86; *p* < 0.001) and sex (Anterior: F_1,18_ = 8.097; *p* = 0.011; Posterior: F_1,17_ = 10.41; *p* = 0.005) in the anterior (Figure 5B) and posterior (Figure 5C) oGCL. In the anterior oGCL, KO mice demonstrated a 98% increase in the total number of Ki67+ compared to WT, with male KO mice exhibiting a ∼246% increase compared to WT males (Sidak’s *post-hoc*; *p* = 0.0005; Figure 5B). In the posterior oGCL, KO mice demonstrated 252% more Ki67+ cells compared to WT mice. Male KO mice had a ∼314% increase compared to male WT, while female WT and KO mice exhibited comparable numbers of Ki67+ (Sidak’s *post-hoc*; *p* = 0.0002; Figure 5C). In the hilus, there was no interaction or main effects of genotype or sex for the total number of Ki67+ cells (Figure 5D). Male KO mice had ∼987% increase compared to WT males (Sidak’s *post-hoc*; *p* = 0.014). However, when divided into anterior and posterior regions, the anterior hilus exhibited a significant interaction (F_1,17_ = 6.767; *p* = 0.019) and effects of genotype (F_1,17_ = 5.653; *p* = 0.029) and sex (F_1,17_ = 5.701; *p* = 0.029; Figure 5E). The posterior hilus had a main effect of genotype (F_1,18_ = 5.625; *p* = 0.0291) but no interaction or effect of sex (Figure 5F). Male KO exhibited a 987% increase in total Ki67+ number as well as an 851 and 981% increase in Ki67+ number in the anterior (Sidak’s *post-hoc*; *p* = 0.003; Figure 5E) and posterior (Sidak’s *post-hoc*; *p* = 0.035; Figure 5F) hilus, respectively, compared to male WT mice. Female WT and KO mice exhibited comparable numbers of Ki67+. In the molecular layer, there was an effect of genotype for the number of Ki67+ in the whole hippocampus (F_1,18_ = 8.671; *p* = 0.009; Figure 5G) as well as the anterior (F_1,18_ = 11.28; *p* = 0.004; Figure 5H) and posterior (F_1,18_ = 7.723; *p* = 0.012; Figure 5I) hippocampus. There was no interaction or effect of sex. Similar to the hilus, male KO mice exhibited significant increases in Ki67+ nuclei compared to male WT across the entire dentate gyrus (150% increase; Sidak’s *post-hoc*; *p* = 0.003) as well as in the anterior (∼124% increase; Sidak’s *post-hoc*; *p* = 0.012) and posterior (∼173% increase; Sidak’s *post-hoc*; *p* = 0.008) dentate gyrus.

When CC3+ cells were quantified in the oGCL (Figures 6A–C), hilus (Figures 6D–F), and Mol layer (Figures 6G–I), only the hilus displayed a significant effect of genotype (F_1,18_ = 8.57; *p* = 0.009; Figure 6D), similar to what was observed at 4 weeks of age (Figure 3D). No main effect of sex or interaction was observed in this subregion. When divided into anterior and posterior regions, only the posterior hilus exhibited a significant main effect of genotype (F_1,18_ = 4.88; *p* = 0.040; Figure 6F), also consistent with what was observed at 4 weeks of age (Figure 3F). Further *post-hoc* analyses revealed that male KO mice exhibited significant increases in CC3+ cells compared to male WT across the entire hippocampus (135% increase; Sidak’s *post-hoc*; *p* = 0.011) as well as in the anterior (193% increase; Sidak’s *post-hoc*; *p* = 0.025) dentate gyrus.

These results show that at 4 weeks of age, the increased proliferation in the dentate gyrus of *Pten* KO mice is largely confined to the neurogenic SGZ (Figures 1–3) but spreads to non-neurogenic regions, including the oGCL, hilus, and molecular layer, by 10 weeks of age (Figures 4–6). In addition, the increased proliferation in the dentate gyrus hilar region was accompanied by a smaller increase in apoptotic cells (Figures 3D, 6D).

### 3.5 Dentate gyrus granule cell volume at 4 and 10 weeks of age

Given that male KO mice had increased proliferation in the neurogenic SGZ at 4 weeks of age and more neuroblasts at 10 weeks of age relative to male WT mice (Figures 1–6), it is possible that these changes in cell number could be due to a greater hippocampal tissue volume. Measuring volume is essential for interpreting changes in cell number (West and Gundersen, 1990; McDonald and Wojtowicz, 2005; Brummelte and Galea, 2010). Volume measurements of the SGZ and GCL combined at 4 and 10 weeks of age are provided in Figures 7A–F and Supplementary Table 3. In brief, *Pten* deletion did not influence volume at either age. These data suggest that the increase in neurogenesis observed in KO mice is an actual change rather than an increase in cell number to match an increase in tissue volume.

### 3.6 Proliferation indices at 4 and 10 weeks of age in the subventricular zone

Adult-born neurons are also produced in the SVZ lining the lateral ventricles, and embryonic deletion of *Pten* has been demonstrated to increase the number of proliferating progenitors in the embryonic telencephalon (Gregorian et al., 2009). Neurons produced in the adult SVZ ultimately migrate along the rostral migratory stream to the olfactory bulb, where they exit the cell cycle and mature into olfactory neurons. To assess if the increase in neurogenesis in male KO mice is restricted to the dentate gyrus, we also examined the number of Ki67+ nuclei lining the SVZ (Figures 8Ai, ii and Supplementary Table 4). At 4 weeks of age, stereological quantification revealed no interaction or effect of genotype or sex (Figure 8B). At 10 weeks of age, there was no interaction or effect of sex, but there was an effect of genotype, indicating a 53% decrease in Ki67+ nuclei in KO mice compared to WT mice (F_1,19_ = 8.415; *p* = 0.009; Figure 8C). Quantification of CC3+ cells (Figures 8Di, ii) revealed no effect of genotype, sex, or interaction at 4 weeks (Figure 8E) or 10 weeks (Figure 8F) of age. These results suggest that *Pten* deletion may first impact dentate gyrus neurogenesis at earlier ages (Figures 1–3) and then progress to other characteristically non-neurogenic regions (Figures 4–6) and the SVZ by 10 weeks of age (Figure 8C).

## 4 Discussion

Based on previous studies (Groszer et al., 2001, 2006; Gregorian et al., 2009), we hypothesized that conditional deletion of the tumor suppressor gene *Pten* in mature granule cells would lead to increased levels of neurogenesis. Using gold-standard markers, unbiased stereological quantification, and analysis of multiple dentate gyrus regions, we report five major findings summarizing how conditional *Pten* deletion affects neurogenesis in the adult hippocampal dentate gyrus (Table 1): (1) in the classical neurogenic dentate gyrus regions (SGZ and GCL), *Pten* deletion leads to a transient increase in proliferating cells at 4 weeks of age and (2) a delayed increase in neuroblasts at 10 weeks of age without observable changes in cell death. Many of these neurogenic changes occurred throughout the dentate septotemporal axis, were specific to males, and were restricted to the dentate gyrus, as (3) an opposite effect was seen in cell proliferation at 10 weeks of age in the SVZ lining the lateral ventricles. In dentate gyrus subfields that are not typically considered to be neurogenic, *Pten* deletion also leads to a (4) male-specific delayed increase in proliferating cells at 10 weeks of age, and (5) region-selective increases in apoptotic cells in the dentate gyrus hilar region. While our data are consistent with earlier studies reporting increased neurogenesis in *Pten* deficient mice, our study adds age-, sex-, and subregion-specific analyses. Together, our findings provide a more comprehensive understanding of the effects of conditional *Pten* deletion on adult hippocampal dentate gyrus neurogenesis, which we discuss in more detail below.

**TABLE 1:** Summary of neurogenesis changes in WT vs. *Pten*^–/–^ mice at 4 and 10 weeks of age.

			Whole Brain		Anterior		Posterior	
			Male	Female	Male	Female	Male	Female
4 Weeks	Ki67	SGZ	64.1	n.s.	133.3	n.s.	119.8	n.s.
		oGCL	n.s.	n.s.	n.s.	n.s.	86.8	n.s.
		Hilus	n.s.	n.s.	129.0	n.s.	n.s.	n.s.
		Mol	n.s.	n.s.	n.s.	n.s.	n.s.	n.s.
		SVZ	n.s.	n.s.	–	–	–	–
	CC3	SGZ	n.s.	n.s.	n.s.	n.s.	n.s.	n.s.
		oGCL	n.s.	n.s.	n.s.	n.s.	n.s.	n.s.
		Hilus	122.0		60.0		n.s.	n.s.
		Mol	n.s.	n.s.	n.s.	n.s.	n.s.	n.s.
		SVZ	n.s.	n.s.	–	–	–	–
	DCX	SGZ/GCL	n.s.	n.s.	n.s.	n.s.	n.s.	n.s.
	Volume	SGZ/GCL	n.s.	n.s.	n.s.	n.s.	n.s.	n.s.
10 Weeks	Ki67	SGZ	n.s.	n.s.	n.s.	n.s.	105.9	n.s.
		oGCL	272.7	n.s.	246.6	n.s.	314.0	n.s.
		Hilus	987.5	n.s.	851.6	n.s.	981.0	n.s.
		Mol	150.0	n.s.	123.8	n.s.	172.8	n.s.
		SVZ	−53		–	–	–	–
	CC3	SGZ	n.s.	n.s.	n.s.	n.s.	n.s.	n.s.
		oGCL	n.s.	n.s.	n.s.	n.s.	n.s.	n.s.
		Hilus	135.0	n.s.	193.3	n.s.	n.s.	n.s.
		Mol	n.s.	n.s.	n.s.	n.s.	n.s.	n.s.
		SVZ	n.s.	n.s.	–	–	–	–
	DCX	SGZ/GCL	98.1	n.s.	98.4	n.s.	90.8	n.s.
	Volume	SGZ/GCL	n.s.	n.s.	n.s.	n.s.	n.s.	n.s.

Percent change in *Pten*^–/–^ vs. WT number of cells immunoreactive for Ki67, CC3, and DCX in the dentate gyrus subregions and subventricular zone lining the lateral ventricles. These summary percentages reflect data presented in Figures 1–8. n.s., no significant change vs. sex-matched WT. –, not quantified. Light-green shading, increased in KO vs. WT. Light-red shading, decreased in KO vs. WT. Mol, molecular layer. oGCL, outer granule cell layer. SGZ, subgranular zone. SVZ, subventricular zone.

The first age analyzed (4 weeks) was selected because this age represents young adulthood in mice. We found more Ki67+ cells in male *Pten*^–/–^ mice at 4 weeks of age throughout the entire septotemporal axis of the SGZ (Figures 1B–D), accordant with the hypothesis that there would be increased proliferation in the dentate gyrus neurogenic niche. We speculate that the increase in Ki67+ SGZ cells is due to increased proliferation of adult-generated dentate gyrus neurons rather than increased proliferation of glial cells due to the regional specificity of the Ki67+ increase. Most glia exist in the hilus and molecular layer and only rarely occupy the SGZ and GCL. Supporting this interpretation, we did not observe any significant increases in Ki67+ number in the oGCL, hilus, and molecular layer at this age (Figure 2). These results agree with the early studies showing that *Pten* deletion leads to increased cell proliferation in the developing cortex (Sun et al., 1999; Groszer et al., 2001, 2006).

While our findings agree with previous studies demonstrating increased proliferation of adult-generated neurons in the hippocampal SGZ (Amiri et al., 2012), there are discrepancies with the Amiri et al. (2012) study regarding the age at which this increase in proliferation occurs. In our study, we observe a transient increase in Ki67+ cell number as early as 4 weeks of age (Figure 1), followed by a return to wild-type levels at 10 weeks of age (Figure 4). Contrarily, Amiri et al. (2012) reported elevated proliferation at 4 months of age that persisted at least until 10 months of age. Several reasons could explain these age discrepancies. There could be compensatory mechanisms, where, in an attempt to control the level of proliferation, additional molecules regulating cell cycle progression are cyclically stimulated and inhibited in response to the early rise in cell proliferation at 4 weeks of age. For example, Hedgehog, Wnt, and Notch signaling pathways (Taipale and Beachy, 2001; Nian and Hou, 2022; Zhang and Beachy, 2023) and cell cycle regulators such as p53, p27^kip1^, p16^INK4a^, and p19^ARF^ promote and inhibit cell cycle progression, respectively, and their levels rise and fall throughout development to tightly control NSPC proliferation. This cyclical stimulation and inhibition of cell cycle molecules could then contribute to age-dependent fluctuations in NSPC proliferation. One of the earliest studies in *Pten*^–/–^ embryos demonstrated that the increased G1 cell cycle progression in *Pten*^–/–^ embryos correlates with downregulation of p27^kip1^ and increased phosphorylation and activation of the kinase PKB/Akt (Sun et al., 1999). Cyclical fluctuations in proliferation levels could also be cell-lineage specific since *Pten* was conditionally deleted in nestin-expressing NSPCs in Amiri et al. (2012), whereas *Pten* was conditionally deleted in GFAP-expressing cells in the current study. Accordingly, the effects of these signaling pathways and cell cycle regulators may depend on the lineage of the NSPCs or the microenvironment in which they reside (Groszer et al., 2001; Yue et al., 2005; Gregorian et al., 2009). There are also methodological considerations, as unbiased stereological quantification was used to obtain an approximation of absolute cell counts in our study, while cell density was obtained by Amiri et al. (2012). Future work entailing a more comprehensive age analysis and subsequent stereological quantification would help address these discrepancies. Supplementing Ki67+ data with an exogenous S-phase marker such as bromodeoxyuridine (BrdU), something that was not done in the current study, would also assess how SGZ cell cycle kinetics is altered in *Pten*^–/–^ mice (Mandyam et al., 2007).

We also analyzed neurogenesis at 10 weeks of age because this allowed us to analyze the progression of neurogenesis as the mice aged. It also requires ∼4−6 weeks in mice for adult-born granule cells to synapse into pre-existing hippocampal networks (Brown et al., 2003; Zhao et al., 2006; Toni et al., 2007; Gu et al., 2012). We also intentionally did not choose a later time point because neurogenesis is known to decline as early as 6 months in mice (Kuhn et al., 1996; Mosher and Schaffer, 2018) and correlates with a decline in cognitive function (Imayoshi et al., 2008), and we did not want advanced age or age-related decline in hippocampal-dependent function to confound our interpretations. Because we observed an increase in Ki67+ cells at 4 weeks of age, we anticipated seeing more DCX+ cells at 10 weeks of age (Figures 4G–I). It is likely that the increased number of DCX+ cells at 10 weeks of age is a result of the 4-week-old Ki67+ cells ultimately differentiating into postmitotic neuroblasts. Our findings also agree with Amiri et al. (2012), where *Pten*^–/–^ mice were reported to have more DCX+ neurons at 4 months of age, although the exact ages that were analyzed are not comparable. It is also possible that the hyperactivity reported in *Pten* knockout mice (Kwon et al., 2006; Ogawa et al., 2007; Amiri et al., 2012; Santos et al., 2017) also contributes to the increased number of DCX+ neurons, particularly given that adult-born neurons are activity-dependent (Deisseroth et al., 2004; Ma et al., 2009; Karadottir and Kuo, 2018) and the hyperexcitable electrophysiological properties of post-mitotic neuroblasts (Liu et al., 2000; Spampanato et al., 2012). Alternatively, the timeline of neuronal differentiation might be altered with *Pten* deletion, and this timeline is dependent on the cell lineage from which *Pten* is deleted since different cell types can have different differentiation timelines. Between our own findings and the findings of Amiri et al. (2012), it is clear that an analysis of additional ages is necessary to obtain a more comprehensive differentiation timeline.

In addition to being hyperexcitable, immature neurons are also susceptible to cell death (Gao et al., 2008). If immature DCX+ cells were susceptible to cell death, we might presume to see fewer DCX+ neurons at 4 weeks of age in *Pten*^–/–^ vs. wild-type mice. However, we found similar numbers of DCX+ (Figures 1H–J) and CC3+ (Figures 1E–G) cells between wild-type and *Pten*^–/–^ mice at 4 weeks of age. Although immature neurons are susceptible to cell death, it is also possible that there could be more apoptosis of Ki67+ cells instead. Regardless of cell type, if there were substantial death of Ki67+ or DCX+ cells, we would also anticipate a smaller GCL at 4 weeks of age; instead, we see a similar dentate gyrus GCL volume at this time point in wild-type and knockout mice (Figure 8). Alternatively, because microglia rapidly clear apoptotic cells within the first few days after birth (Sierra et al., 2010), it is possible that not all apoptotic cells were captured in our analysis. Future studies examining additional ages would provide a greater understanding of the relationship between the number of hyperexcitable DCX+ neurons and cell death in *Pten*^–/–^ mice.

Because dentate gyrus granule cells are mainly glutamatergic, the increased proliferation at 4 weeks of age in *Pten*^–/–^ mice could contribute to increased network activity (Zhang et al., 2012; Zhou et al., 2019). This is supported by studies showing that mice missing *Pten* develop spontaneous seizures at about 13 weeks of age and increase in severity as they age (Kwon et al., 2006; Ogawa et al., 2007; Amiri et al., 2012; Santos et al., 2017). Recent research shows that seizures are not merely recurring spurts of electrical activity in large neuronal populations. Instead, smaller networks of multiple neural cell populations and circuits within the dentate may be hyperconnected to each other, thereby promoting seizure-like activity (Morgan and Soltesz, 2008; Bui et al., 2015; Sparks et al., 2020). These hyperconnections could be magnified with the increased number of adult-born glutamatergic neurons in *Pten*-deficient mice.

In addition to the highly neurogenic SGZ layer, we also found increased Ki67+ cells in hippocampal subregions traditionally not thought to have neurogenic potential (Figure 5). We specifically observe a statistical increase in Ki67+ cells in the hilus, oGCL, and molecular layer of male *Pten*^–/–^ mice throughout the entire septotemporal axis at 10 weeks of age. These ectopic cells could be a result of increased excitatory synaptic input as described above, or it could be a result of dividing glia. Supporting the latter, astrocytes and microglia are more abundant in the hilus and molecular layer and it is becoming increasingly clear that glial cells can influence hippocampal neurogenesis (Cope and Gould, 2019). Moreover, oligodendrocyte progenitor cells are the most widespread proliferating cell type in the brain and could also explain the increase in cell proliferation (Dawson et al., 2003). Of particular interest, microglia have been shown to regulate multiple stages of adult neurogenesis, including proliferation, differentiation, and survival of NSPCs in healthy and epileptic brains (Luo et al., 2016a). Microglia can also modulate the appearance of ectopic granule cells. In the healthy brain, microglia primarily clear apoptotic granule cells via phagocytosis (Sierra et al., 2010), but after seizure activity, activated microglia will phagocytose granule cells that do not express cleaved caspase-3 (Luo et al., 2016b). In contrast, other studies report that genetic deletion of microglia or attenuation of microglial activation reduces the number of granule neurons, indicating that microglia might also promote seizure-induced neurogenesis (Ali et al., 2015; Mo et al., 2019). Regardless of whether microglia restricts or accelerates seizure-induced neurogenesis, it is increasingly clear that microglia residing outside of the SGZ can heavily influence neurogenesis levels following seizure activity. We have recently shown that *Pten* deletion leads to increased numbers of microglia in the cortex, hippocampus, and cerebellum (Narvaiz et al., 2022), although it is not known which hippocampal subregion displays the increased numbers of microglia. In our current study, because we observe a region-selective increase in CC3+ apoptotic cells in the hilus (Figures 3D–F, 6D–F), it is possible that the increased number of Ki67+ and CC3+ cells could be due to changes in microglia. Because dividing cells in various subfields of the dentate gyrus have different cell cycle kinetics (Mandyam et al., 2007), additional analyses of glial markers in conjunction with cell cycle markers in multiple dentate gyrus regions in both wild-type and knockout tissue at multiple ages are essential to elucidate the role of proliferating glia.

In addition to proliferating Ki67+ cells, post-mitotic DCX+ cells have been documented outside the SGZ (Shapiro et al., 2008, 2011). Amiri et al. (2012) also observed an increase in mossy fiber thickness and elongated dendrites in the molecular layer of 4 and 7-month-old *Pten*^–/–^ mice. In the current study, DCX+ cells in the oGCL were sparsely populated; therefore, we combined DCX+ cell counts in the SGZ and GCL. DCX+ cells in the hilus and molecular layer were also difficult to detect and precluded us from enumerating these cells outside of the SGZ and GCL. Future studies using a more precise method, such as transgenic DCX+ reporter mice or labeling mitotic cells, may more reliably display DCX+ neuroblasts outside the SGZ.

While we report that *Pten* deletion leads to successive changes in proliferating cells and neuroblasts, we find no change in the volume of the dentate gyrus GCL (Figure 7; Supplementary Table 3, and Table 1). Volume is important to consider since an increase in the number of cells could simply be due to an increase in structure volume. However, our data indicate there is no change in the size of the GCL. Additionally, many studies examining neurogenesis following seizures report cell counts in terms of density, not total cell number as determined stereologically. Reporting both volume and total cell counts is important, as increased cell density could be from either an increase in the number of cells or a decrease in tissue volume (West and Gundersen, 1990). Therefore, our data indicating increased neurogenesis with no change in GCL volume implies an actual increase in neurogenesis.

In addition to the SGZ, the SVZ is also a neurogenic niche that primarily generates new neurons that ultimately integrate into the olfactory bulb (Lim and Alvarez-Buylla, 2016). To elucidate if *Pten* deletion in granule cells would also impact SVZ proliferation, we also enumerated Ki67+ cells in this region. In contrast to our SGZ findings, we observed an overall decrease in the number of dividing Ki67+ cells in 10-week-old KO mice (Figure 8C). One reason for these different outcomes may be that these two regions produce different types of neurons with different functions. In addition, the production of adult-born neurons seems to serve very different roles in the adult hippocampus and olfactory bulb. In the olfactory bulb, adult neurogenesis is responsible for replacing granule neurons and is essential for olfactory bulb maintenance and function (Goldman, 1998). In contrast, adult neurogenesis in the hippocampus adds new neurons (rather than replacing them) and is critical for the growth of the dentate gyrus (rather than dentate gyrus maintenance) (Imayoshi et al., 2008). Our findings also contradict previous work where conditional deletion of *Pten* in adult NSPCs in the SVZ increased stem cell self-renewal and expansion of NSPCs and was associated with increased size and mass of the olfactory bulb (Gregorian et al., 2009). However, data from Gregorian et al. (2009) are difficult to interpret because *Pten*^–/–^ mice also exhibit more GFAP + cells, a larger SVZ volume, and larger olfactory bulb mass. Therefore, it is difficult to interpret if the increased proliferation is simply due to an increase in glial proliferation, SVZ size, and/or olfactory bulb mass.

As a result of our current study, we propose several important future directions. First, it is not yet known how *Pten* deletion influences the fate of adult-born cells in the dentate gyrus. Conflicting studies either show that cell fate commitments of *Pten*^–/–^ NSPCs are largely undisturbed in the embryonic cortex (Groszer et al., 2001, 2006) or demonstrate increased differentiation of adult-born SGZ cells into S100B + astrocytes (Amiri et al., 2012), premature astrocytic differentiation of radial glial cells (Bonaguidi et al., 2011), and premature differentiation of Bergmann glia in the cerebellum (Yue et al., 2005). While the increase in SGZ-located Ki67+ cells in our study likely reflects an increase in adult-born granule neurons, as opposed to glia, determining the fate of dividing SGZ cells will necessitate phenotypic analysis of proliferating SGZ cells with a neuronal marker and/or a study of electrophysiological properties. Second, as other seizure models result in abnormal migration of adult-born dentate gyrus neurons (Dashtipour et al., 2003; Shapiro et al., 2008, 2011; Murphy et al., 2012) and PTEN has been shown to be essential for cell migration and lamination of the cerebellum (Marino et al., 2002; Yue et al., 2005), future work could assess whether the greater number of Ki67+ and CC3+ cells in hilus and oGCL indicates abnormal migration. In our study, DCX+ staining outside the SGZ and GCL was difficult to quantify, but future work using virally-labeled DCX+ cells or neuronal phenotyping could be informative (van Praag et al., 2002; Laplagne et al., 2006; Wood et al., 2011; Kathner-Schaffert et al., 2019; Cole et al., 2020). Third, it will be important to consider if *Pten* deletion changes the dendritic morphology of the adult-born dentate gyrus granule cells as has been reported in other CreER^T2^ lines, including Nestin-creER^T2^ (Amiri et al., 2012) and Gli1-creER^T2^ (Santos et al., 2017). In this study, we were not able to definitively quantify the arborization of DCX+ processes due to the high density of DCX+ dendrites that are typically found in mice (Wu and Hen, 2014). To address this, future studies could utilize retroviral labeling to more sparsely label dentate gyrus neurons, allowing for a more conclusive analysis of DCX+ morphology.

Lastly, we consistently observed male-specific changes in dentate gyrus neurogenesis of *Pten* KO mice. The reason for this remains unknown, partly due to the large but mixed literature on sex-related differences in seizure activity and epilepsy. For example, spontaneous generalized epilepsy is predominant in females, while region-specific epilepsy occurs more frequently in males (Reddy, 2014; Samba Reddy, 2017). While the physiological mechanisms underlying these differences are unknown, factors such as neurotransmitter systems, steroid hormones, and sexual dimorphism of neural circuits may underlie these sex-dependent differences (Reddy, 2014; Samba Reddy, 2017). Research using various *Pten* Cre lines has also demonstrated mixed sex-specific circuit hyperexcitability in KO mice (Santos et al., 2017; Molinaro et al., 2023), and much of this variability likely depends on physiological factors such as steroid hormones and glutamatergic-dependent signaling. Sex-dependent differences in neurogenesis have also been documented, with evidence suggesting that males may be more susceptible to stress [reviewed in Yagi and Galea (2019)]. While hypothetical at this time, if males tend to demonstrate changes in neurogenesis in response to stress [reviewed in Yagi and Galea (2019) and references therein], then it is conceivable that we observed male-specific trends in our study because *Pten* KO mice are susceptible to developing seizures (Backman et al., 2001; Kwon et al., 2001, 2003; Ogawa et al., 2007; Ljungberg et al., 2009; Santos et al., 2017). Because sex-dependent differences within the brain have become increasingly crucial for the treatment of neurological disorders, additional investigation into sexually dimorphic effects is warranted.

## Data availability statement

The original contributions presented in the study are included in the article/Supplementary material, further inquiries can be directed to the corresponding author.

## Ethics statement

The animal study was approved by the Baylor University Institutional Animal Care and Use Committee and the National Institutes of Health Guidelines for the Care and Use of Laboratory Animals. The study was conducted in accordance with the local legislation and institutional requirements.

## Author contributions

SL: Conceptualization, Data curation, Formal analysis, Funding acquisition, Investigation, Methodology, Project administration, Resources, Supervision, Validation, Visualization, Writing – original draft, Writing – review & editing. BL: Data curation, Formal analysis, Methodology, Writing – review & editing. PW: Methodology, Writing – review & editing. KB: Methodology, Writing – review & editing. JL: Conceptualization, Formal analysis, Funding acquisition, Investigation, Methodology, Project administration, Resources, Supervision, Validation, Writing – review & editing.

## Supplementary material

Supplementary table 1 Statistical results for cell quantification (Ki67+, CC3+, and DCX+ cells) and SGZ/GCL volume measurements in the dentate gyrus at 4 weeks of age. Sexes were analyzed separately (via 2-way ANOVA) and combined (via two-tailed unpaired *t*-test).

Supplementary table 2 Statistical results for cell quantification (Ki67+, CC3+, and DCX+ cells) in the dentate gyrus at 10 weeks of age. Sexes were analyzed separately (via 2-way ANOVA) and combined (via two-tailed unpaired *t*-test).

Supplementary table 3 Statistical results for SGZ/GCL volume measurements in the dentate gyrus at 4 and 10 weeks of age. Sexes were analyzed separately (via 2-way ANOVA) and combined (via two-tailed unpaired *t*-test).

Supplementary table 4 statistical results for cell quantification (Ki67+ and CC3+ cells) in the subventricular zone at 4 and 10 weeks of age.
